# Role of the Nuclear Lamina in Age-Associated Nuclear Reorganization and Inflammation

**DOI:** 10.3390/cells9030718

**Published:** 2020-03-14

**Authors:** Lidya Kristiani, Miri Kim, Youngjo Kim

**Affiliations:** Department of Integrated Biomedical Science and Soonchunhyang Institute of Medi-Bioscience, Soonchunhyang University, Cheon-an 31151, Korea; lidya.k94@gmail.com (L.K.); kimmiri0715@gmail.com (M.K.)

**Keywords:** nuclear lamina, lamins, aging, cellular senescence, chronic inflammation, 3D chromatin structure, Hi-C

## Abstract

Aging is characterized by the gradual loss of tissue function and integrity. Activation of inflammatory responses accelerates the deterioration of cells and tissues. Many studies have shown that alteration of the components of the nuclear lamina is associated with inflammation, both in vivo and in vitro. However, the mechanism by which the nuclear lamina regulates inflammation is largely unknown. Recent studies have suggested that the nuclear lamina regulates both organization of the three-dimensional chromatin structure at the nuclear periphery and global gene expression, such as the expression of inflammatory response genes. Here, we discuss the current updates in the research on nuclear lamina alteration, activation of inflammation, and nuclear reorganization in models of cellular senescence and organismal aging.

## 1. Introduction

Human biological aging represents progressive cell deterioration, impaired tissue functions, defense system malfunctions owing to stressors, and decreased ability to maintain homeostasis [1]. Although a definitive mechanism of aging has not yet been established, the widely accepted theories surrounding aging include oxidative stress, mitochondrial dysfunction, glycation, immune system deregulation, and progressive telomere shortening. While these models partially explain some aspects of aging, its cellular and molecular mechanisms remain largely ambiguous. Many studies have shown that chronic inflammation is associated with the acceleration of biological aging [2]. Chronic inflammation is characterized by the production of low-level proinflammatory cytokines in the circulatory system without obvious external infections. This leads to constitutive low-level activation of the immune system, resulting in systemic tissue damage [3]. This phenomenon is often referred to as “inflammaging” [4].

Inflammaging is associated with age-associated diseases and is a significant risk factor for morbidity and mortality in aged humans. The most prevalent chronic diseases associated with inflammaging include the following: cardiovascular diseases such as atherosclerosis, stroke, and heart failure [5]; cancer [6,7]; metabolic diseases such as type II diabetes [8,9]; sarcopenia and muscle impairments [10,11,12]; osteoporosis [13,14]; neurological diseases such as Alzheimer’s disease, Parkinson’s disease, and dementia [15,16]; and frailty [17,18]. Epidemiological studies have commonly detected mild and persistent elevation of inflammatory biomarkers such as interleukin 6 (IL-6) and C-reactive protein (CRP) [19]. These markers predict the incidence of the aforementioned age-associated conditions. Furthermore, recent genome-wide association studies revealed that genetic factors involved in regulating inflammatory pathways are associated with human longevity [20]. However, it is still unclear whether the mild elevation of inflammatory pathways causes age-associated diseases, or whether chronic inflammation is a secondary consequence from their onset. It is possible that positive feedback between chronic inflammation and age-associated diseases activates both processes, rather than a single overarching factor being responsible. Paradoxically, an acute and high level of inflammation protects the body from harmful conditions caused by damaged tissues or pathogenic infection [21]. Exhaustive eradication of the entire inflammation network would destroy immune homeostasis and defense systems against infections. Understanding the distinct factors regulating chronic and acute inflammatory pathways is necessary to alleviate age-associated deterioration without disrupting the metabolic balance between inflammatory and anti-inflammatory pathways.

Multiple mechanisms have been suggested to lead to chronic low-level inflammation upon aging. These include oxidative stress [22], glycation [23], mitochondria dysfunction [24,25], immunosenescence [26,27], chronic infection [28,29], hormonal changes [30,31], lipid metabolism and obesity [32,33], diet [34], renin-angiotensin system (RAS) [35,36], epigenetic modification [37,38], cellular senescence and SASP (senescence associated secretory phenotype) [39], and telomere dysfunction [40]. However, the molecular triggers of age-associated chronic inflammation are still poorly understood. Alteration of the nuclear lamina (NL), a protein meshwork composed of lamin intermediate filaments, is implicated in the development of chronic inflammation upon aging. The NL is also critical for building and maintaining the three-dimensional chromatin structure and epigenetic landscapes. Understanding how NL alteration influences nuclear organization and the gene expression network, in addition to how it initiates chronic inflammation, is crucial. This would elucidate how impairment of the aforementioned pathways is coupled with the initiation of low-level inflammation. Furthermore, this would provide a foundation upon which to develop clinical reagents for treating age-associated abnormalities via modulating chronic inflammation. Although the means by which the nuclear lamina regulates 3D genome organization is well described in many other review articles, its functional significance has not been thoroughly discussed, particularly in the context of cellular and organismal aging. In this review, we discuss how recent findings have begun to reveal the functions of lamins as genome organizers, coupling aging and chronic inflammation.

## 2. The Nuclear Lamina

Eukaryotic nuclei are surrounded by the nuclear envelope (NE), separating the nucleoplasm from the cytoplasm. The NE is formed by the NL and two juxtaposed lipid bilayers: the inner nuclear membrane (INM) and the outer nuclear membrane (ONM). The NL is located underneath the inner nuclear membrane (INM) and is composed of a fibrous protein meshwork and nuclear membrane-associated proteins [41]. The NL primarily provides the nuclear membranes with structural integrity, and lamins are the major constituents of the NL. Immunocytochemical analyses revealed that lamin isoforms are classified into A- and B-type lamins [42]. In mammals, the three lamin genes *LMNA, LMNB1*, and *LMNB2* all encode different lamin isoforms. A-type lamins such as lamin A, C, AΔ10, and C2 are encoded by the single gene *LMNA*, whereas B-type lamins are encoded by *LMNB1* and *LMNB2*. *LMNB1* encodes lamin B1, and *LMNB2* encodes lamins B2 and B3. An evolutionary study of lamins suggested that all metazoan cells have at least one B-type lamin, and *LMNB1* is the most ancient and conserved form of the lamin gene [43].

Lamins are classified as type V intermediate filaments. In vitro studies of lamin assembly revealed that parallel coiled-coil interactions of lamin monomers at the rod domains form dimers, which in turn form a head-to-tail polymer [44]. Lamin polymers are assembled into a half-staggered paracrystalline array through lateral association. In vitro studies showed that excess lamins could rapidly form 10-nm-wide intermediate filaments [45,46]. However, analysis by cryo-electron tomography (ET) revealed that A- and B-type lamins assemble into 3.5-nm-thick filaments in vivo, which comprises at least two head-to-tail polymers in cross-section [47]. These filaments display a high degree of flexibility in their length, which ranges from 50–2700 nm, and are packed into a 14 ± 2-nm-thick layer. Although A- and B-type lamin dimers can co-assemble to form lamin filaments in vitro [48], an ex vivo study suggested that endogenous A- and B-type lamins form separate filaments [49]. Indeed, genetic deletion experiments suggested that A- or B-type lamins can form fairly normal filaments in the absence of other types of lamins [50,51]. In addition, a recent study using lamin-depleted somatic cells showed that when alone, each isoform allows even NL organization and a normal distribution of nuclear pore complexes at a sufficiently high concentration [52]. Despite functional overlapping, A- and B-type lamins contribute to different aspects of the biophysical and mechanical properties of the NE in somatic cells. A-type lamins are responsible for the varying levels of nuclear stiffness in different cell types, while B-type lamins confer elasticity to the NE [53,54,55,56,57]. Although A- and B-type lamins are functionally separated and form distinct filaments, they mutually affect their expression and organization [58]. Interestingly, changes in the ratio of A- to B-type lamins lead to alterations in migration properties and somatic cell integrity [59,60,61].

The clinical significance of the NL was determined in the 1990s after intensive genetic mapping and sequencing. Over 180 mutations in genes encoding lamins are associated with human diseases referred to as laminopathies [62]. Among these are autosomal dominant Emery–Dreifuss muscular dystrophy (EDMD), limb girdle muscular dystrophy, dilated cardiomyopathy, heart-hand syndrome, restrictive dermopathy, mandibuloacral dysplasia, lipodystrophy, Charcot–Marie–Tooth disease, and Hutchinson–Gilford progeria syndrome (HGPS) [63]. *LMNA*-associated diseases are characterized by a wide spectrum of clinical manifestations and frequent overlapping features. However, accumulating genetic evidence has determined connections between specific mutations in distinct *LMNA* domains and different diseases [62]. There is no known human disease associated with the loss-of-function mutation in *LMNB1*. *LMNB1* gene duplication causes adult-onset autosomal dominant leukodystrophy (ADLD), characterized by CNS demyelination with a chronic multiple sclerosis-like phenotype [64]. *LMNB2* point mutations cause progressive myoclonic epilepsy and partial lipodystrophy [65,66].

While A-type lamins are rarely expressed in pluripotent stem cells or embryos during early development [67,68,69], at least one B-type lamin is expressed in most embryonic and somatic cell types. Immune cells have unique profiles of lamin isoforms. Lamin A/C is rarely detected in naïve T cells in human peripheral blood lymphocytes (PBLs) and mouse splenocytes, but lamin A/C expression levels dramatically increase upon T cell activation [70]. Conversely, mature neutrophils have very low levels of lamin A/C and lamin B1, but a moderate level of lamin B2 [71]. As B-type lamins are implicated in many basic cellular processes, such as DNA replication, transcription, and cell division, it is widely accepted that B-type lamins are essential for cell survival and proliferation [72]. However, mouse embryonic stem cells (mESCs) lacking either B-type lamins (*LMNB1* and *LMB2*) or all lamins (*LMNB1*, *LMNB2* and *LMNA*) survive and proliferate [73,74]. Mice with depleted *LMNB1* and *LMNB2* developed to term [73], and *LMNB1* and *LMNB2* tissue-specific deletion in keratinocytes and hepatocytes had no effect on cell proliferation [51,75]. These observations suggest that B-type lamins are not required for essential cellular functions. Instead, analyses of B-type lamin knockout mice revealed that *LMNB1* and *LMNB2* have specific developmental roles in multiple tissues, including brain, lung, and bone tissue [73,76,77].

The first and widely recognized *LMNA* knockout mouse with an exon 8–11 deletion dies at approximately 8 weeks of age with signs of cachexia, muscular dystrophy, and cardiomyopathy [50]. The more recent *LMNA* knockout mouse has blocked expression of exons 2–12 via exon 1 deletion. These mice die 16–18 days after birth, suggesting that *LMNA* knockout mice with an exon 8–11 deletion have a hypomorphic phenotype [78,79]. In addition to originally reported muscle defects, recent studies using *LMNA* knockout mice with different deletion alleles revealed that these mice also have impaired lipid metabolism via the mTOR signaling pathway [79,80]. Numerous *LMNA* knock-in mice have been generated to replicate *LMNA*-associated human diseases. In contrast to humans, most mice harboring human disease-causing *LMNA* mutations exhibit partial or no phenotypes in a heterozygous state. However, these mice develop disease-like phenotypes in a homozygous state, which is not fully understood [81]. Nonetheless, these mouse models provide critical insight into the etiology of *LMNA*-associated diseases and the connections between specific *LMNA* mutations and diseases.

## 3. Fluctuation of Lamin B1 Level and Aging

Evidence has shown that differential lamin B1 expression is closely related with age-associated disorders via evolution, and most studies emphasize the importance of lamin B1 reduction in aging phenotypes.

In *Caenorhabditis elegans*, the nuclear architecture in most non-neuronal cell types (muscle, skin, and intestine cells) is progressively and stochastically changed 8 days post-hatch, as determined by observing changes in nuclear shape and heterochromatin loss [82]. The deterioration rate of the nuclear architecture was influenced by the insulin/insulin-like growth factor 1 (IGF-1)-like signaling pathway, which has a major role in regulating the lifespan of worms. Long-lived *daf-2* (homolog of IGF-1) mutant worms exhibited retarded alteration of nuclear shape, whereas the short-lived *daf-16* (homolog of FoxO) mutant worms showed accelerated nuclear shape changes. Although lamin protein reduction was not observed in normal aged worms, specific reduction of *lmn-1* (the sole lamin B1 isoform in worms) by RNAi during postembryonic development significantly shortened their lifespan. This suggests that B-type lamins are required for normal longevity [82].

**Figure 1:**
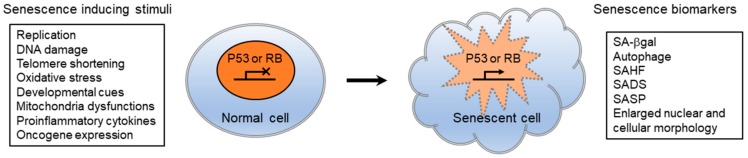
Senescence-inducing stimuli and biomarkers of cellular senescence. Diverse stresses/stimuli cause irreversible cell cycle arrest and cellular senescence. Senescence pathways are associated with two distinct modes of senescence referred to as replicative senescence (RS) and oncogene-induced senescence (OIS), depending on senescence inducing stimuli. Although RS and OIS share some common downstream pathways and biomarkers, they also have substantial differences. SA-βgal, senescence-associated β-galactosidase; SAHF, senescence-associated heterochromatin foci; SADS, senescence-associated distension of satellite DNA sequences; SASP, senescence-associated secretory phenotype.

Cellular senescence is often referred to as replicative senescence and is a potent defense mechanism against tumor formation. The process irreversibly halts the proliferation of cells that are likely to undergo malignant transformation [83]. It is characterized by cell cycle arrest, proinflammatory cytokine secretion, enlarged and abnormal nuclear morphologies, and chromatin rearrangement. These are typically mediated by the p53 and pRB tumor suppressor pathways (Figure 1). The human diploid fibroblast (HDF) cell line WI-38 progressively underwent replicative senescence after 39 passages of subculture, which was identified by observing senescence-associated β-galactosidase (SA-β-gal) induction, lack of BrdU incorporation, and senescence-associated heterochromatin foci (SAHF) formation [84]. When WI-38 cells entered replicative senescence, lamin B1 transcripts and proteins were dramatically reduced, while lamin B2 or lamin A/C levels remained unchanged. Oncogenic Ras expression in WI-38 cells induced premature senescence, leading to lamin B1 reduction via an Rb-dependent pathway. Conversely, RNAi mediated *LMNB1* knockdown in WI-38 cells slowed cell proliferation and induced premature cellular senescence phenotypes. Both p53 and Rb pathways were required for the *LMNB1* knockdown-induced senescence phenotypes in WI-38 cells, while only the p53 pathway was required for defective proliferation. Interestingly, cells with proliferation defects displayed a p53-dependent reduction in mitochondrial reactive oxygen species (ROS) shortly after *LMNB1* silencing. The generation of modest levels of ROS during normal cell cycle progression is known to positively contribute to the proliferation rate [85]. Thus, it is feasible that decreased ROS level upon *LMNB1* silencing is a molecular trigger for defective proliferation and senescence phenotypes in human fibroblasts. Other studies showed that lamin B1 reduction is a hallmark of human fibroblasts and keratinocytes undergoing replicative senescence in vitro, as well as normally aged human skin [86,87].

Studies of cells cultured in vitro have anticipated that lamin B1 reduction contributes to age-associated tissue and organ malfunction in vivo. The influence of age-associated lamin B1 reduction in different tissues was thoroughly explored in *Drosophila*, a relatively simple and short-lived model. Chen et al. found that LAM, the fly ortholog of lamin B1, was dramatically decreased in the fat body and brain upon aging, while LAM levels were unchanged in the gut and heart [88,89]. Surprisingly, the effect of LAM reduction in fat body cells was not limited to the fat body itself but led to midgut hyperplasia via systemic inflammation. The complexity of mammalian tissues makes it difficult to estimate the exact role of lamin B1 reduction in tissue dysfunction and aging. Recently, the impact of age-associated lamin B1 reduction on tissue function and integrity in mammalian tissues has begun to be elucidated. The vertebrate thymus is a relatively simple organ that mainly consists of thymocytes and thymic epithelial cells (TECs). The second phase of thymic involution and size reduction, typical characteristics of aged thymus, are closely associated with the functional decline of the mammalian immune system [90]. Yue et al. showed that lamin B1 decreased by 65% in TECs from 20-month-old mice compared to that in TECs from 2-month-old mice, while the levels were maintained in the thymocytes [91]. Moreover, lamin B1 reduction in TECs kinetically correlated with the gradual decrease of thymic T cell numbers in all tested time periods. TEC-specific lamin B1 deletion resulted in thymocyte reduction in the thymus, suggesting that lamin B1 reduction in TECs upon aging can lead to decreased naïve T cell production in the thymus. In vivo lamin B1 reduction was also observed in mammalian tissues treated with senescence-inducing stimuli. Mice treated with a senescence-inducing dose of ionizing irradiation had significantly decreased levels of lamin B1 protein and mRNA in the liver [87]. In the lungs, cellular senescence is implicated in chronic obstructive pulmonary disease (COPD). Cigarette smoking (CS) is one of the leading risk factors for COPD development. A mouse model of COPD showed that long-term (6 months) CS exposure leads to COPD with cellular senescence phenotypes in the airways [92]. Saito et al. showed that lamin B1 levels decreased in lung airway epithelial cells in long-term CS-exposed mice but not in alveolar epithelial cells. Similar lamin B1 reduction was observed in lung and cultured human bronchial epithelial cells (HBECs) in patients with COPD. Although lamin B1 is ubiquitously expressed, the effects of lamin B1 reduction occurred in a cell or tissue-specific manner in both flies and mice. It is possible that actively proliferating cell types quickly replace damaged or senesced cells, making it difficult to detect lamin B1 reduction. Taken together, lamin B1 reduction or loss may be a common component of senescence pathways both in vitro and in vivo.

In contrast, Autosomal Dominant Leukodystrophy (ADLD) and ataxia telangiectasia (AT) are linked with increased lamin B1 expression [64,93]. Some studies explored how elevated lamin B1 levels have a role in the development of these human diseases. Studies revealed that lamin B1 overexpression induced cellular senescence and impaired proliferation, whereas shRNA-mediated lamin B1 depletion inhibited proliferation but did not induce cellular senescence in human fibroblasts [86,93]. Interestingly, lamin B1 overexpression phenotypes were exacerbated with lamin A/C reduction, which is accompanied by G1 arrest and telomere-associated DNA damage. Based on these results, the authors concluded that increased lamin B1 expression leads to cellular senescence by inducing telomeric DNA damage. These studies suggest that stoichiometric changes in NL composition are associated with the onset of pathological conditions and aging in various human tissues.

## 4. Alteration of Lamin A/C and Aging

The *LMNA* gene is a hotspot for disease-causing mutations. It has received much attention owing to its association with a variety of diseases, collectively called laminopathies. One of the most devastating laminopathies is Hutchinson–Gilford Progeria Syndrome (HGPS), a very rare genetic disorder with clinical features of premature aging in humans. The disease is diagnosed soon after birth with a range of pathological characteristics, including hair loss, growth retardation, loss of subcutaneous fat, weakened muscle function, osteoporosis, and bone hypoplasia [94,95]. Moreover, lamin B1 reduction was observed in human fibroblasts derived from patients with progeria [86]. Over 90% of patients with HGPS harbor a de novo single-base substitution, G608G (GGC > GGT), within exon 11 of *LMNA*. This mutation creates a new lamin A splicing isoform with an internal 50 amino acid deletion lacking a ZMPSTE24 cleavage site, but not affecting the C-terminal CaaX motif. As a consequence, the mutant prelamin A, termed progerin, becomes permanently farnesylated and carboxymethylated [96,97]. Since the cellular defects observed in HGPS overlap with those that occur during physiological aging, HGPS models have been powerful tools to identify and characterize the cellular and molecular mechanisms underpinning normal aging. Moreover, the relevance of HGPS to physiological aging is evident, as small amounts of progerin are also present in the cells of normally aged individuals [98]. The shared mechanisms between HGPS and normal aging include impaired DNA repair, reduced telomere length, epigenetic landscape alteration, dysregulation of IGF1 signaling, decreased sirtuin and AMPK activities, mitochondrial dysfunction, proteolysis reduction, increased cellular senescence, and decreased stem cell regenerative capacity [99].

Lamin A/C levels are increased in the epididymal white adipose tissues (eWAT) of obese mice and humans [100,101]. Obesity induces chronic inflammation in adipose tissues, contributing to the development of insulin resistance and type 2 diabetes. Lamin A/C is specifically enhanced in CD11c^+^ M1 adipose tissue macrophages (ATMs) in obese mice, while lamin A/C depletion in macrophages abolished chronic inflammation and obesity-mediated metabolic disorders in vitro and in vivo [100]. Examining whether an increase in lamin A/C in M1 ATMs also occurs in lean adults upon aging would be an interesting avenue of research, in addition to whether it contributes to a higher prevalence of metabolic imbalances in older adults.

## 5. NL Alteration Is Associated with Chronic Inflammation

Aging processes are characterized by the gradual loss of tissue function and integrity, which is linked to various age-related diseases. Low-grade and chronic inflammation has been shown to exacerbate dysfunction of various organs and contribute to disease development upon aging [102]. The relationship between NL deterioration and induction of chronic inflammation has been demonstrated. Many senescent cells and tissues are known to secrete proinflammatory cytokines, growth factors, and proteases (referred to as SASP), which induce chronic inflammation. A study using cultured cells showed that reduction of lamin B1 levels (2 days) by senescence-inducing stimuli occurred much earlier than the induction of SASP and other cellular senescence phenotypes (10 days) [87]. RNAi-mediated lamin B1 reduction was sufficient to develop such phenotypes [84]. These results suggest that decreased lamin B1 has a crucial role in the onset of senescence-associated events, including SASP.

A recent study using *Drosophila* showed a potentially causal relationship between lamin B1 reduction and chronic inflammation in vivo [88]. In *Drosophila*, the reduction of LAM in fat bodies upon aging is associated with upregulation of the immune deficiency (IMD) signaling pathway. This is equivalent to the mammalian TNFα signaling pathway. RNAi-mediated LAM knockdown in young flies clearly induced inflammation in fat bodies. Both age-associated and RNAi-mediated LAM reduction led to the upregulation of immune response genes by disrupting the structure of heterochromatin. LAM reduction, therefore, has a causative role in age-associated inflammation in *Drosophila* fat bodies. In aged flies, inflamed fat bodies secrete antimicrobial peptidoglycan recognition proteins (PGRP) into circulating hemolymph. This is equivalent to the mammalian blood system, and the secretion leads to systemic chronic inflammation and causes hyperplasia in the midgut. RNAi-mediated LAM depletion in the fat bodies also caused systemic inflammation and gut hyperplasia. Conversely, proinflammatory cytokines trigger lamin B1 reduction in aged cells and tissues. In aged mouse thymus, lamin B1 reduction in TECs is linked to the induction of proinflammatory cytokines such as TNF-α, IL-1α, IL-1β, and IL-6 in thymic immune cells [91]. When primary TECs isolated from young mouse thymus were treated with proinflammatory cytokines, all cytokines except for IL-1α induced lamin B1 reduction and cellular senescence within 5 days after treatment. This suggests that upon aging, proinflammatory cytokines secreted from senesced thymic immune cells trigger lamin B1 reduction in TECs and thymic dysfunction.

Conversely, lamin A/C upregulation in ATMs was associated with adipose tissue inflammation and insulin resistance in obese mouse models [100]. Lamin A/C overexpression in cultured macrophages activates IKKβ, induces NF-κB nuclear translocation, and activates ATMs. Elevated lamin A/C levels are also associated with ERK1/2 signaling during T lymphocyte activation [70]. Lamin A/C deficiency in naïve T cells leads to enhanced regulatory T-cell differentiation but reduces Th1 polarization via epigenetic regulation of T-bet, the Th1 master regulator [103]. This suggests that lamin A/C upregulation has a crucial role in triggering inflammatory responses in immune cells. Enhanced NF-κB activity also occurs upon progerin expression in epidermal cells in normal and progeroid mouse models [104,105]. A recent study showed that progerin induces replication stress and a cell-intrinsic innate immune response [106]. Single-molecule replication analysis (DNA fiber assays) revealed that ectopic progerin expression in retinal pigment epithelial (RPE) cells induces DNA replication fork stalling and nuclease-mediated degradation, causing replication stress. Progerin-induced replication stress activates a STAT1-mediated interferon (IFN)-like response via the cCAG/STING pathway. Reducing replication stress and the IFN-like response by treating progeroid cells with calcitriol ameliorates their aging phenotypes. In contrast to ATMs, wild type lamin A overexpression in RPE does not cause replication stress or an IFN-like response.

## 6. Senescence-Associated Nuclear Reorganization and the NL

Senescent cells undergo profound changes in their chromatin structure and architecture. These changes have been attributed to a key component of cellular senescence [107]. Classic cytological studies have formed our understanding of chromatin structures in senescent cells. Genotoxic stresses activate the DNA damage response (DDR), which is associated with a series of chromatin unfolding and restoration processes to maintain genome integrity. DDR-mediated chromatin decondensation and recondensation typically consist of chromatin remodeling, incorporation of histone variants, non-core histone protein recruitment, and covalent core histone modification [108]. The most prominent senescence-associated core histone modification is phosphorylation of the histone H2A variant H2AX (γH2AX) by ATM/ATR kinases. γH2AX holds the free ends of DNA and recruits DNA repair proteins at double-strand breaks (DSBs). In response to DNA damage, γH2AX spreads several mega-bases surrounding DSBs and aggregates to form microscopically discernable foci. However, γH2AX foci are predominantly found in irradiation-induced senescent cells but are rarely detected in senescent cells induced by other stimuli. Instead, oncogene-induced senescent cells are generally associated with senescence-associated heterochromatin foci (SAHF), visible by DAPI staining. SAHFs are densely stained chromatin domains detected in the nucleus and are associated with high-mobility group A (HMGA) proteins, the histone variant mH2A, and HP1 proteins [109]. Further analyses revealed that SAHFs lack nascent transcripts and active histone modification marks, such as H3K4me3 and H3K9Ac, but are enriched for heterochromatin marks such as H3K9me3 [109], H4K20me3 [110] and H3K27me3 [111]. However, as with γH2AX foci, SAHFs are not universally detected in all types of senescent cells.

Recent studies have shown global changes in the epigenomic landscape of senescent cells. DNA methylation and chromatin accessibility assays revealed contrasting changes in euchromatin and heterochromatin regions, which may lead to interrupted gene expression upon senescence. While gene-poor constitutive heterochromatin regions displayed increased chromatin accessibility and decreased DNA methylation levels, some regions of gene-rich euchromatin had decreased chromatin accessibility and increased DNA methylation levels upon replicative senescence [112]. Altered histone occupancy also contributes to senescence-associated chromatin changes. Cells undergoing replicative senescence were found to have a global decrease of the core histone H3, H4, and the linker H1. However, it is still unclear whether these changes result from histone protein expression or the eviction and subsequent degradation of these proteins.

DamID and ChIP-seq assays identified lamina-associated domains (LADs) ranging from 0.1 to 10 MBp in size [113,114]. Albeit not completely overlapped, the vast majority of LADs share their genomic and epigenetic features with heterochromatin [114]. Mapping LADs in mouse ESC-differentiated cells revealed that gradual and cumulative LAD pattern alteration is correlated with reprogramming the gene expression network during lineage commitment and terminal differentiation [115]. These results led to the idea that age-associated NL alteration may influence gene expression by affecting global 3D chromatin structures, especially in heterochromatin regions. Indeed, NL alteration causes large-scale nuclear landscapes. The loss of both lamin B receptor (LBR) and lamin A/C, essential components of the NL in most somatic cells caused detachment of peripheral heterochromatin into the nucleus, forming an inverted nuclear architecture [116]. Lamin B1 knockdown in human fibroblasts resulted in global changes in the chromatin landscape, as detected in replicative senescent cells. These changes included increased H3K4me3 and H3K27me3 in LADs and H3K27me3 depletion in inter-LADs and euchromatin regions [38]. Lamin B1 depletion also led to H3K9me3-marked heterochromatin redistribution from the nuclear periphery, facilitating SAHF formation in the nucleus [117].

Development of high-throughput chromosome conformation capture (Hi-C) has allowed the development of a high-resolution map of the 3D chromatin structure. Based on Hi-C maps of human and mouse cells, it was revealed that chromatin is hierarchically organized into distinct domains at different scales [118,119]. In the largest scale, chromosomes form different chromosome territories (CT) within interphase nuclei. Genomic regions with similar transcriptional activity and epigenetic features in a CT tend to colocalize, forming discrete active A and inactive B compartments. Within each compartment, chromatin is further organized into topologically associated domains (TADs) and sub-TADs. Hi-C with a high sequencing depth can finally visualize long-range chromatin interactions from 10–100 kb in size.

Hi-C has begun to visualize changes in the 3D chromatin structure in cells with altered NL. HGPS skin fibroblasts were first analyzed by Hi-C to evaluate the importance of the NL and chromatin organization in progerin-induced cellular deteriorations [120]. Active A and inactive B compartments are sharply demarcated in normal cells. However, the boundaries between A and B compartments became faint in late-passage HGPS cells. Furthermore, a subset (12%) of A and B compartments eventually underwent compartment switching. The loss of compartmentalization in heterochromatin regions was correlated with alteration of H3K27me3 deposition and DNA-lamin A/C associations, in addition to differential gene expression in HGPS cells. This suggests that progerin accumulation at the nuclear periphery disrupts the association of heterochromatin regions with the NL. This leads to changes in the 3D chromatin structure in these regions, as shown by compartment shifts. Heterochromatin reorganization also occurs in oncogene-induced lamin B1-reduced cells [121]. Microscopic observation has shown that heterochromatin regions are detached from the nuclear periphery and form dense SAHFs in the nucleus. Conversely, Hi-C revealed decreased chromatin interactions within each heterochromatin domain. However, interactions between different heterochromatin domains increased in senescent cells, which may explain the microscopic detection of SAHFs as dense chromatin spots.

**Figure 2:**
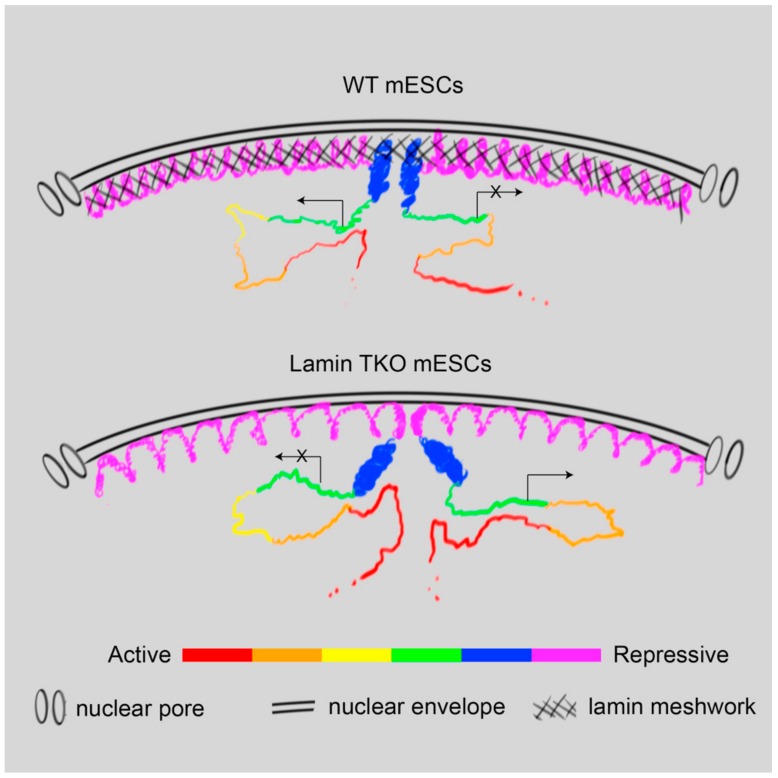
A lamin meshwork-cage model. Lamin loss leads to the decondensation of constitutive heterochromatin, characterized by HiLands-P or cLADs (purple). This may push facultative heterochromatin characterized by HiLands-B or fLADs (blue) away from the nuclear periphery. Detached HiLands-B or fLADs undergo compartment shifts. These changes disrupt 3D chromatin structures in both heterochromatin and the neighboring euchromatin, leading to alteration of global gene expression. Figure is from Zheng et al. 2018 [123], courtesy of the authors.

Hi-C assays of senescent and progeroid cells provide insight into how the NL influences 3D chromatin organization and subsequent gene regulation. Despite this, aging processes have made it difficult to determine whether these are directly caused by NL alteration, or whether they are secondary consequences of other senescence-induced signaling pathways. LADs can be divided into two different types of domains, based on their behavior during cell differentiation or underlying genomic and epigenetic features [115,122]. LAD mapping in different cell types revealed that constitutive LADs (cLADs) maintained their interactions with the NL in all tested cell types, whereas facultative LADs (fLADs) displayed cell type-specific interactions [115]. Combinatorial analyses of lamin B1 binding, histone occupancy, and epigenetic marks divided LADs into HiLands-B and -P, which largely overlap with fLADs and cLADs, respectively [122]. Therefore, to explore the role of the NL in 3D genome organization, examining how the two different types of LADs are altered in a system depleted of all lamins is essential. Genome-wide analysis of the 3D chromatin structure using Hi-C, DamID, and FISH showed that lamins differentially regulate the two types of LADs in mESCs [123]. In lamin-null mESCs, constitutive heterochromatin characterized by HiLands-P or cLADs displayed increased inter-TAD interactions and expanded their volume. Simultaneously, their association with the nuclear periphery was maintained or slightly increased, as demonstrated by emerin DamID. Conversely, facultative heterochromatin characterized by HiLands-B or fLADs was detached from the nuclear periphery and underwent a compartment shift upon lamin loss, such that the compartment indices shifted from B toward A. Finally, lamin loss-mediated 3D chromatin structure alteration was correlated with differential transcription in both heterochromatin and neighboring euchromatin. Based on these results, the authors proposed a “lamin meshwork-cage model” explaining how lamins regulate the 3D chromatin structure and global gene expression network (Figure 2).

Based on the “lamin meshwork-cage model,” the NL may regulate the 3D chromatin structure either at or near immune response gene loci, in addition to their gene expression. A chromatin-lamina interaction map of a variety of cells indicated that immune response genes are enriched in LADs [73,115,124]. The NL may therefore silence immune response genes by locating them at the nuclear periphery, maintaining their heterochromatin properties in young or normal states. In aged or senescent cells, NL alteration may decondense or release the immune response genes from the nuclear periphery, leading to derepression of those genes. Although the concept that the NL functions as a genomic organizer to regulate the 3D chromatin structure and gene expression is attractive, other models to elucidate how NL alteration leads to aging phenotypes should not be ignored. The NL is connected to the cytoskeleton via the nucleoskeleton and cytoskeleton (LINC) complex, which allows the transmission of mechanical force from the cell surface to the nucleus [125]. Depletion or mutation of NL constituents and components of the LINC complex impairs nucleo-cytoskeletal force transmission and mechanosensitive gene expression, which is discussed extensively elsewhere [126,127]. As shown in progerin-expressing cells, alteration of NL constituents or integrity may also lead to DNA damage, which in turn activates immune responses such as the NFκB and IFN-like signaling pathways.

## 7. Conclusions

Growing evidence suggests that cellular senescence and organismal aging are associated with profound changes in NL components, with lamin B1 reduction being predominant. NL alteration appears to have a causative role in activating inflammatory responses, as demonstrated both in vitro and in vivo. It is also feasible that a subtle deterioration of the NL initiates low-level inflammatory responses, along with other age-associated cellular responses. This leads to further alteration of the NL via a positive feedback loop. Structural changes in chromatin organization appear to be at least partly responsible for age-associated cellular responses, including activation of inflammatory response genes. High resolution mapping of 3D chromatin structures has revealed that the high-order structure of heterochromatin regions displays profound alterations in senescent or progeroid cells, as seen by compartment shifts and detachment from the nuclear periphery. Genomic analyses using lamin-null embryonic stem cells showed that the NL has a fundamental role in maintaining the 3D chromatin structure and global gene expression. However, there are still many questions to be addressed. The mechanism by which high-order chromatin structures influence individual chromatin interactions, such as long-range interactions between enhancers and promoters, remains unclear. It is also important to identify the specific long-range chromatin interactions responsible for age-associated cellular defects. It is still unclear whether lamin isoforms have specific roles in regulating the 3D chromatin structure or whether the NL as a whole is crucial for maintaining it. Finally, it is important to explore the 3D chromatin structure of senescent cells in vivo. As most genomic techniques require millions of cells, this is currently challenging.
